# Not all plasma cells are made equal: well-hidden layers of heterogeneity

**DOI:** 10.3389/fimmu.2026.1809241

**Published:** 2026-05-11

**Authors:** Audrey Anoh Akessé, Amélie Bonaud

**Affiliations:** 1 CNRS UMR7276/INSERM U1262, University of Limoges, CRIBL lab, Limoges, France; 2 Institut de Recherche Omega Health, Limoges, France

**Keywords:** B cell, heterogeneity, humoral immune response, immune response, plasma cell

## Abstract

Plasma cells (PCs) are the final stage of B cell development and sustain long-term humoral immunity by continuously secreting antibodies. Once considered a homogeneous population defined by a short-lived versus long-lived dichotomy, PCs are now recognised as highly heterogeneous. Recent advances have overturned several dogmas, revealing that long-lived plasma cells (LLPCs) can arise from diverse B cell precursors, and persist in tissues beyond the bone marrow. PC heterogeneity is shaped by intrinsic factors, including B cell origin, antigen affinity, BCR signalling strength and immunoglobulin isotype, as well as extrinsic factors such as tissue-specific microenvironments, cytokines and cellular interactions at induction and maintenance sites. Furthermore, temporal variables, termed “Moment”, including age, sex, and inflammatory status, modulate PC fate throughout their maturation. This process also plays a central role in the emergence of heterogeneity within these LLPCs. Together, these parameters define a dynamic, context-dependent PC landscape with significant implications for immune regulation and vaccine design.

## Introduction

Plasma cells (PCs) represent the terminal stage of B cell differentiation and serve as the principal effectors of humoral immunity. Through the sustained production and secretion of antibodies, they ensure long-term protection against pathogens and contribute to immune memory. The differentiation of B cells into PCs is notably orchestrated by a tightly regulated network of transcription factors, including BLIMP-1, XBP1, and IRF4, accompanied by profound morphological and metabolic adaptations that support their high-rate secretory activity (1–4). While short-lived PCs emerge rapidly in secondary lymphoid organs following antigen exposure, long-lived PCs (LLPCs) migrate and predominantly reside in the bone marrow, maintaining antibody titers for years or even decades (5–7). These empiric definitions are now partially redefined, and we will implement them in light of recent knowledge about these cells. Beyond their protective roles, perturbations in PC development, survival, or function underlie a range of pathological conditions, including autoimmune diseases, immunodeficiencies, and PC malignancies such as multiple myeloma. A comprehensive understanding of PC biology, from differentiation and maintenance to the regulation of function, remains crucial for the rational design of strategies aimed at modulating antibody responses in both health and disease.

For many years, PCs remained relatively unexplored, their existence inferred primarily from the detection of circulating antibodies, indirect evidence of their presence somewhere within the organism. Although their capacity for antibody production has long defined their identity, the mechanisms by which antibodies are secreted remain incompletely understood. Whether antibodies exit the cell through passive diffusion across the plasma membrane, vesicular transport, or alternative routes is still debated, suggesting that multiple parallel processes may operate to preserve this essential function (8–11). Their rarity and technological limitations have led to a lack of study, resulting in the persistence of dogmas that have been deconstructed one after another in recent years;

1st dogma: PC heterogeneity is limited by a simple dichotomy; short or long lived
2nd dogma: LLPCs derive from germinal centre (GC) B cells
3rd dogma: LLPCs produce switched immunoglobulin (Ig)
4th dogma: LLPCs are only found in the bone marrow, in fixed niches

Recent advances in high-throughput and single-cell technologies have revealed that PCs are far from a homogeneous population. Numerous studies, summarized in several comprehensive reviews, converge on the same conclusion: PCs display remarkable molecular, phenotypic, and functional diversity (4, 6, 12, 13). Early transcriptomic analyses had long reinforced the notion of PCs as a uniform entity, a view that is not entirely correct. Although distinct PC subsets have now been described, they appear to share several core characteristics, the most prominent being the extreme enrichment of Ig transcripts. Approximately 60–70% of the total PC transcriptome is composed of Ig mRNAs, which tend to mask other transcriptional variations, particularly in single-cell studies (14–16). Owing to this overwhelming abundance of Ig sequences, the first evidence of heterogeneity among PCs emerged largely from studies comparing the isotypes of antibodies they produce. Subsequent investigations have revealed that PCs secreting distinct Ig isotypes also exhibit differences in phenotype, transcriptome, and function. For instance, membrane Ig expression is partially retained in subsets producing IgM or IgA, suggesting a continuum between antibody-secreting cells and their B cell precursors (17–19). Moreover, several genes involved in cell adhesion and migration, such as *ITGA4* and *ITGAL* encoding VLA-4 and LFA-1, respectively, are differentially expressed according to the antibody isotype (20–24). These molecular differences have been further supported by *trans*-interactome analyses, which identified specific interaction networks between PCs and their surrounding stromal or immune partners, suggesting that distinct survival niches may exist for IgM-, IgG-, and IgA-producing PCs (25). Among these subsets, IgA-secreting PCs represent a striking example of tissue adaptation. These cells express the chemokine receptor CCR10 on their surface, a feature consistent with their preferential localization, or possible relocalization to mucosal tissues and the skin (26–28).

Such findings raise important and still open questions: does the origin of a PC determine its fate and longevity, both in terms of its B cell precursor and its anatomical site of differentiation? Furthermore, could the route of antigen entry and the nature of the immune context influence PC heterogeneity? In the following sections of this review, we aim to dissect the determinants of PC heterogeneity, exploring how it emerges during B cell activation, how it is shaped by the local tissue environment, and how it evolves during PC maturation and maintenance.

## Intrinsic factors influencing PC heterogeneity

The generation of heterogeneous PC populations begins at the earliest stages of B cell activation, depending of their location, nature and type of activation. Both the cellular origin of the B cell (e.g. follicular B cell, marginal zone B cell, GC B cell or memory B cell) and the nature of the antigenic stimulus (T cell-dependent versus T cell-independent), together with the influence of B cell receptor (BCR) co-stimulatory signals, shape subsequent differentiation trajectories and contribute to PC heterogeneity. For a long time, it was described that marginal zone (MZ) B cells preferentially gave rise to short-lived PCs, whereas follicular B cells, through GC formation, were prone to differentiate into PCs producing highly mutated and class-switched antibodies, thus considered the only cells capable of becoming LLPCs. However, this model was refined several years ago by the demonstration that non-mutated LLPCs can also derive from MZ B cells (14, 15, 29–32). These observations have led to a new perspective, which is now considered to be a contextual plasticity and probabilistic fate. The long-standing dogma regarding the kinetics of extrafollicular versus GC responses remains largely unchanged: extrafollicular responses are rapid, whereas GC formation requires a delay, except in memory responses. Nevertheless, the concept of response longevity has evolved (12). It was long thought that the onset of the GC marked the termination of the extrafollicular response; it is now clear that this response, often masked by the GC reaction, can persist for several weeks or even months (7, 33, 34). At later stages of the GC response, when B cells face the decision to differentiate into either memory B cells or PCs, antibody quality and affinity maturation again play a central role (34–37). Competitive mechanisms that assess antibody affinity dictate the signalling inputs received by B cells, notably through the MYC axis (38–42). B cells that have achieved less affinity improvement preferentially differentiate into memory B cells, whereas those with the highest affinity differentiate into PCs (43–46). The biological rationale is that memory B cells, upon antigen re-exposure, can re-enter GCs and further refine their antibodies if required, whereas PCs must already possess the highest-quality antibodies (47). However, several recent studies have nuanced this view by showing that GCs are progressively re-invaded by naïve B cells, a process that is essential to sustain competition within these structures, leading to highly varied levels of affinity (48, 49). This has been demonstrated both in vaccination models (e.g. NP-OVA) involving injections and in viral infections (e.g. SARS-CoV-2 or influenza) administered intranasally (48, 49). Timestamping experiments have further demonstrated that LLPCs are generated throughout the entire immune response, and that hypermutated and class-switched LLPCs are over-represented, likely as a consequence of prolonged GC longevity (7, 15, 33, 50, 51). As a result, PCs expressing antibodies of varying affinities are generated, suggesting a substantial degree of affinity-based heterogeneity among PCs, all of which retain the potential to become LLPCs.

Another critical parameter governing PC differentiation is the strength of BCR signalling. As early as 2013, using the murine LMP2A model to analyse the impact of strong versus weak BCR signalling, we demonstrated that weak BCR signalling favours PC differentiation, whereas strong signalling promotes B-cell maintenance (52), in agreement with the “less is more” hypothesis of C. Goodnow (53). Although these studies are open to criticism due to the artificial nature of the model, they had the advantage of dissociating signal strength from signal identity. Since then, multiple studies have reached similar conclusions. Recently, it has been shown that IgG1-expressing B cells display lower surface BCR levels than IgM/IgD expressing B cells and exhibit a greater propensity to differentiate into PCs (54). Within GCs, such differences in BCR signalling strength, due to the amount of crosslinked BCR mobilised, may arise through asymmetric cell division (55). *In silico* studies further support the hypothesis that the amount of antigen retained in association with the BCR is a key determinant of PC differentiation (56). Indeed, asymmetric divisions generate imbalances that result in daughter cells with fewer BCR-antigen complexes at the cell surface, accompanied by altered distributions of B-cell transcription factors, thereby enabling the activation of the PC transcriptional programme.

Collectively, these studies indicate a probable influence of immunoglobulin isotype and antigen specificity on PC differentiation. Moreover, antigen properties, including their capacity to co-induce Toll-Like Receptor (TLR) activation, are likely to further shape underlying PC heterogeneity, as demonstrated in Khamyath et al. *(available on bioRxiv)* (57). Recently, we have shown that antigen nature conditions the emergence of distinct PC subpopulations, distinguishable by differential expression of surface markers (57).

In conclusion, PC heterogeneity is determined both by the identity of the B cell that gives rise to the PC and by the nature of the antigen and the signalling pathways it elicits. PC heterogeneity therefore depends, directly or indirectly, on the antigen itself, which ultimately orchestrates the immune response as a whole. Beyond antigen nature, the route of entry and, consequently, the anatomical site of immune induction also play a crucial role, as each site provides a distinct microenvironment that favours the emergence of PC heterogeneity.

## Extrinsic factors influencing PC heterogeneity

Localisation implicitly refers to differences in microenvironments. Such differences are multifaceted. Firstly, it is important to distinguish between the induction site and the maintenance site, as these are not necessarily identical. Secondly, at both the induction and maintenance sites, an inflammatory and/or infectious microenvironment is superimposed on the tissue-specific microenvironment, depending on the context. The PC compartment is established and organised by B cell responses and differentiation, which are influenced by distinct cellular components and cytokine contexts found in these environments.

The diversity of induction sites has been comprehensively reviewed by D. Tarlinton, who emphasised that PC heterogeneity is essential to generate the most effective immune response according to anatomical location, both in terms of antigen specificity and longevity, well described in human (13). For example, the significance of IgA responses in mucosal surfaces, such as those found in the skin, the gastrointestinal tract, and the female reproductive tract, can be accentuated (26, 28, 58, 59). These sites are in immediate proximity to mucosal surfaces and the external environment. Beyond IgA production, which enables continuous sensing of potential pathogenic intrusions, PCs in these tissues may contribute to the establishment of an anti-inflammatory microenvironment through the production of IL-10, a cytokine essential for preventing immune overactivation at these major entry sites for pathogens (59, 60). Through these regulatory and modulatory functions, PCs represent key cellular actors within these tissues.

The generation of such PCs at their sites of induction is directly shaped by the local cellular composition, notably through interactions with innate immune cells, either directly or indirectly via the prevailing cytokine context. The influence of natural killer T (NKT) cells on B cell activation and, ultimately, PC differentiation has been well documented (61, 62). More recently, invariant NKT (iNKT) cells have been shown to differentiate into iNKT follicular helper–like cells (iNKTfh) implicated in controlling GC B cell responses. This reciprocal regulation arises from a tri-cellular dialogue between Tfh cells, iNKTfh cells and B cells, and dysregulation of any of these three components favours indirectly the emergence of autoantibodies and, consequently, pathogenic PC subsets (63). In the context of pulmonary viral infections, additional regulatory actors include distinct populations of memory B cells. *Bona fide* lung-resident memory B cells (CXCR3^+^) respond in an antigen-specific manner, in contrast to bystander memory B cells (CXCR3^-^), which do not mount antigen-specific responses (64). Although these different cellular players clearly contribute to shaping the response, the respective contributions of direct versus indirect interactions remain incompletely elucidated. In particular, the impact of cytokine production, especially IL-4 and IL-21, on B cell heterogeneity and the resulting PC populations is not always fully accounted for. The observed differences may be due to artefacts resulting from the experimental models used to activate B cells. Until recently, the majority of studies investigating B cell responses and/or PC differentiation relied on well-established but artificial models, such as NP-KLH or defined murine peptide antigens, which ultimately more closely mimic vaccination than natural infection. The difference between these two contexts has always been unclear and still is in some situations. However, a major difference exists between vaccination and infection, namely the degree and nature of the pro-inflammatory microenvironment and inflammation itself. These two parameters critically influence PC differentiation and the durability of humoral responses, thereby revealing the existence of heterogeneous PC populations (34, 65–68).

Vaccine development is a continuously evolving field, both in terms of vaccine platforms (protein-based, DNA, RNA, etc.) and adjuvant formulations. However, vaccine efficacy, particularly with regard to the longevity of humoral immunity, is often assessed primarily through correlations between the presence of antigen-specific antibodies and the presumed presence of antibody-secreting cells, with little or no direct investigation of these cells and/or their heterogeneity. Nevertheless, several recent studies have highlighted the importance of the vaccination route. Intramuscular administration, traditionally employed, predominantly induces systemic immune responses, whereas respiratory delivery elicits strong local immunity in addition to systemic responses, thereby conferring superior protection while simplifying the administration procedure (69). The nature of the PCs generated by these distinct routes remains to be fully characterised. It is crucial to understand the differences between PCs that stay in the place where they were first created, and then move to the bone marrow, and those that only go to the bone marrow (70, 71). This is important because it shows that there are different types of PCs. In order to control how many are produced and ensure they continue to function well over a long period of time, it is necessary to understand these differences. In light of recent work from the group of A. E. Denton and M. A. Linterman, it appears that a complete and qualitatively comparable GC response can be generated within tertiary lymphoid structures (TLS), a situation that may also apply to nasal vaccine–induced immune responses (72). While intramuscular injection minimally stimulates the draining lymph nodes and supports the establishment of bone marrow–resident memory, recent advances in vaccinology increasingly challenge this route of administration (69, 73).

The site of maintenance of LLPCs was long considered to be exclusively the bone marrow. It is now clear that this view is incomplete. Bone marrow niches are the most extensively characterised environments supporting long-term LLPC survival. Historically, they were regarded as the exclusive locations for this. Several reputable reviews have provided detailed information on their composition (74, 75). Key shared components include CXCL12-producing stromal cells and sources of survival factors, such as APRIL and IL-6, via eosinophils and macrophages (76–78). The functional redundancy of these cells has called into question the exclusive role previously attributed to eosinophils (79, 80). Direct cell–cell interactions mediated through specific signalling axes are also essential. Although these niches were long thought to be highly stable and uniform, we and others have demonstrated differences in niche interactions depending on the Ig isotype produced, revealing previously unappreciated heterogeneity among bone marrow niches (25, 81). Another recently overturned dogma is the notion that the bone marrow is a static environment in which PCs remain immobile within a single niche for their entire lifespan. Instead, PCs can migrate within the bone marrow and may even exit this compartment (50). Rare PCs in the bone marrow, once envisaged as solitary cells occupying individual niches, appear to persist more efficiently within clusters in which APRIL plays a critical role (82). The existence of such clusters raises the possibility of direct PC–PC interactions and regulatory mechanisms, as isolated PCs exhibit reduced survival and appear less capable of becoming LLPCs (21). Whether this reflects an intrinsic inability to acquire long-lived status or a lack of appropriate receptors required for essential interactions remains unresolved. Importantly, the bone marrow is not the only site of PC maintenance. There is an associated induction site for every effector site where PCs are required. We now know that this interconnection also allows LLPCs to be maintained at these sites. PCs can thus persist in the genital tract, lacrimal glands, meninges and skin, as well as in the bone marrow (13). The spleen, particularly the red pulp, also serves as a maintenance site for LLPCs, and it has recently been reported that IgE-producing LLPCs preferentially persist in this organ (83). However, LLPCs resident in the spleen have a shorter lifespan than those typically found in the bone marrow (15). Unsurprisingly, these newly identified maintenance sites display highly distinct cellular and cytokine compositions, strongly suggesting site-specific regulation of PC longevity that is likely linked to their specialised functions. Furthermore, the interplay between maturation state and microenvironmental cues amplifies PC heterogeneity. PCs at different stages of maturation express distinct combination of receptors, including those for survival cytokines and adhesion molecules, exhibit divergent metabolic profiles, such as differences in mitochondrial activity and adaptation to hypoxia and consequently display differential sensitivity to niche-derived signals (4, 12, 14). Whether these alternative maintenance sites support the completion of PC maturation remains an open question. Traditionally, PC maturation was thought to be completed exclusively within the bone marrow; it is now unclear whether this dogma still holds. Several questions remain unanswered: do all PCs possess the intrinsic capacity to complete maturation at their site of induction, or is this ability restricted to those fated to become LLPCs? Given that PC mobility is not limited to immature cells, do must PCs transit through the bone marrow to mature before relocating to other effector sites? Finally, do the structural and molecular characteristics of each effector site provide specific signals that actively shape or generate PC heterogeneity? Collectively, these questions remain open and represent key challenges for the field.

## Discussion

As discussed above, our understanding of PCs is evolving rapidly and substantially, with significant changes in both their emergence and maintenance. Long-standing paradigms are being progressively challenged, and PC heterogeneity, which has long gone unnoticed due to the technical limitations imposed by their scarcity, has now emerged as an undeniable yet poorly understood feature. The parameters governing the establishment of this heterogeneity are numerous, and several groups have already proposed models highlighting the combined importance of intrinsic factors related to PCs and their B cell precursors, as well as extrinsic factors linked to the microenvironment at both their sites of generation and long-term maintenance (13, 75). Together, these already extensive parameters contribute to shaping PC heterogeneity. A growing number of markers, described by independent and non-overlapping studies, collectively support this emerging framework. For example, CXCR3, MHC class II, and TIGIT are preferentially associated with short-lived PCs, whereas EpCAM, Ly6a, and CD93 are more commonly linked to LLPCs (7, 15, 84). At present, researchers have not clearly established their function and mainly use them as markers. However, in the case of CXCR3, for example, its absence is inversely proportional to an increase in Blimp1 expression (15). EpCAM, on the other hand, appears to be essential for anchoring IgM- or IgG-producing plasma cells in their niche (15). Finally, while the mechanism associated with CD93 remains unclear, its absence compromises the maintenance of LLPCs (84). Additionally, graded patterns of protein expression, such as the progressive loss of MHC class II and/or Slamf6, are associated with PC maturation and longevity (85). In our recent work, we further identified CD62L as a marker of immaturity, and demonstrated that combinatorial expression gradients of CXCR4, FcγRIIb, and CD93 are critical for the establishment of T-dependent and T-independent PC immune responses (57). The significance of CD138 for function has been highlighted by studies from Dr. Fooksman’s group, despite it having been viewed in the past as simply a reference PC marker. Their findings demonstrate that PCs expressing high levels of CD138 display a selective advantage in terms of survival, medullary clustering, and retention within the bone marrow (82, 86, 87). Notably, intrinsic and extrinsic parameters converge when considering adhesion molecules and chemokine receptors or ligands, which are expressed by PCs themselves yet directly mediate interactions with the microenvironment. Distinguishing the relative contributions of intrinsic and extrinsic components could be extremely informative. Is PC heterogeneity the result of both factors? Or is it predominantly mediated by one of them? Is cellular plasticity possible? Transfer experiments, in which PCs generated at one induction site are reintroduced to another, could allow us to track their subsequent evolution, particularly to determine whether the generated heterogeneity remains the same.

**Figure 1:**
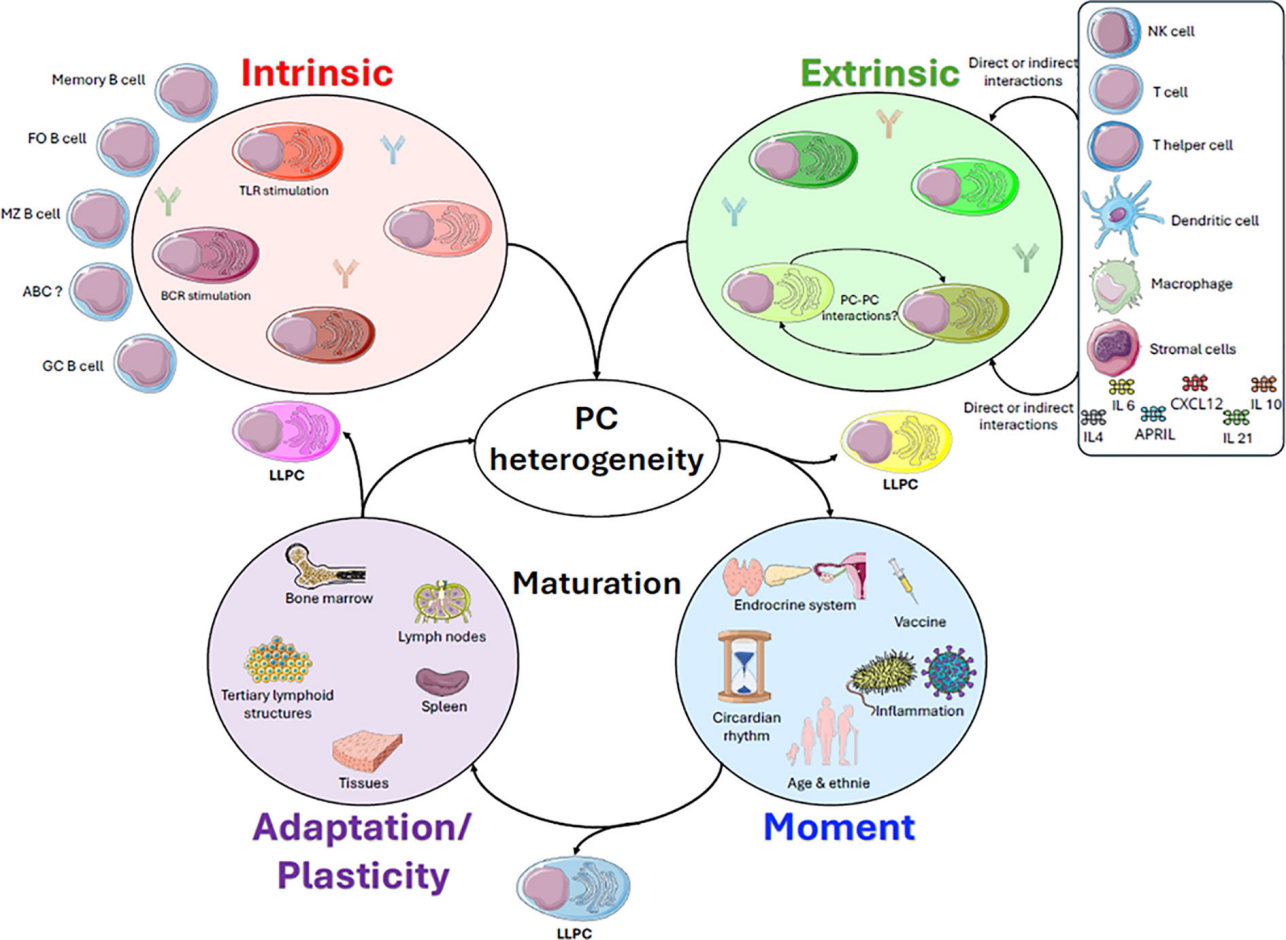
The dynamic regulation of plasma cell heterogeneity. Plasma cell (PC) heterogeneity is a highly complex and interconnected landscape. There are many factors that monitor this heterogeneity. Here, we focus on five of the most important: intrinsic, extrinsic, tissue adaptation, maturation and temporal factors. Intrinsic factors begin at the earliest stages of B cell activation, when different types of B cell encounter antigens, particularly follicular B cells (FO B cells), marginal zone B cells (MZ B cells), memory B cells and ageing B cells (ABC). The nature of the antigenic stimulus dictates the type of PC that is generated, whether T cell-dependent with the B cell receptor (BCR) or T cell-independent with toll–like receptor (TLR). Extrinsic factors are linked to the induction, microenvironment and maintenance of PCs. The generation of PCs at their sites of induction is directly shaped by the local cellular composition, notably through direct and/or indirect interactions. This includes innate immune cells such as natural killer (NK) cells, T cells, T helper cells, dendritic cells and PCs, as well as cytokines such as IL-10, IL-21 and IL-4. PC maintenance requires key components such as stromal cells that produce CXCL12 and sources of survival factors such as APRIL and IL-6 via eosinophils and macrophages. PC heterogeneity is essential for generating the most effective immune response according to anatomical location, in terms of both antigen specificity and longevity. PC sites are closely linked to extrinsic factors and include secondary lymphoid organs, tertiary lymphoid structures, and sites distinct from lymphoid tissues, such as the meninges, skin, and gut, as well as bone marrow. These sites display highly distinct cellular and cytokine compositions, suggesting that PC longevity is regulated in a site-specific manner, which is likely to be correlated with their specialised functions. The fourth parameter is a highly variable factor depending on an individual’s condition at a given moment in time. It encompasses both inflammatory contexts (bacteria or viruses) and characteristics specific to the individual, such as sex, age and ethnic background. Hormonal context and circadian rhythms are also part of this temporal factor. Incorporating PC maturation characteristics into this model provides a unifying biological process that links initial heterogeneity, tissue localisation, and temporal context within a single, dynamic framework governing PC fate. Their emergence depends on successful maturation. Together, these complementary factors monitor PC heterogeneity and influence PC fate, and it is probably their implication that favours the generation of long-lived PCs that sustains long-term humoral immunity.

Despite the intricacies of this model, which include numerous parameters, it seems overly generalised in its approach. It fails to consider “moment”, that is the unique characteristics and variations that occur across individuals’ lifetimes (**Figure 1**). Parameters that are inherent to each individual include sex and the associated hormonal context, ethnic background and HLA haplotype for example (88). Indeed, sex-based differences in immune responses and autoimmune disease susceptibility highlight intrinsic heterogeneity linked, at least in part, to hormonal context. Similarly, the COVID-19 pandemic clearly revealed differential antiviral responses across ethnic groups, potentially reflecting variations in HLA-mediated antigen presentation (83). Experiments on mice could provide answers to these questions by comparing the heterogeneity of PCs in pregnant and non-pregnant mice, or in animals that have undergone ovariectomy. The results could then be compared with those from castrated versus non-castrated male mice to illustrate the importance of sex hormones in shaping PC heterogeneity. Such analyses could be accompanied by approaches such as fate mapping to assess survival, measuring CD138 marker density, monitoring CXCR4 or CD93 expression gradients and evaluating potential effects on cellular metabolism. Temporal variability also represents an important factor. At the daily scale, circadian rhythms have a strong influence on immune efficiency, affecting both vaccine responses and immunotherapeutic outcomes. Investigating the role of genes that control circadian regulation in PC heterogeneity following vaccinations administered at different times of the day would help clarify this influence (89, 90). Immune responses also differ substantially across the lifespan (91, 92). For example, infants with immature immune systems mount distinct responses to those of adults or elderly individuals. Although immune ageing is well characterised, its impact on PC heterogeneity is less clear. LLPCs generated decades earlier can persist; however, the properties of newly generated PCs in aged individuals remain poorly understood (58, 93). Environmental factors, including pollution, tobacco exposure and alcohol consumption, can also influence immune responses by promoting chronic inflammatory states, which are associated with an increased risk of autoimmune diseases. It is likely that these environmental pressures influence PC heterogeneity, and this could be investigated through single-cell epigenetic profiling following exposure to such factors.

Ultimately, the maturation model is probably central to the heterogeneity of LLPCs. Their emergence depends on the success of the maturation process, the duration of the immune response and the tissue microenvironment(s) involved (**Figure 1**). The maturation model considers both intrinsic factors, such as the origin of the B cell and variations in early maturation events that determine initial maturation potential, and extrinsic signals, such as cytokine contexts and the distinct environments of induction sites that either promote or arrest maturation (14, 94). This leads to a spectrum of PC states. This maturation framework also explains how PCs persist in non-medullary tissues, thereby supporting the idea that LLPCs can exist outside the bone marrow. All inter- and intra-individual variables encompassed within the “moment” regulate the pace, trajectory, and completion of PC maturation. Chronic inflammation or ageing may shift PCs towards partially mature or dysfunctional states. Successful LLPC establishment appears to be based on the temporal dimension and longevity of the maturation process. Recent findings further suggest that PCs should not be considered fixed terminal endpoints, but rather cells that undergo progressive maturation following differentiation (14, 94). From this perspective, the maturation model provides a unifying biological process that links initial heterogeneity, tissue localisation and temporal context within a single, dynamic framework that governs PC fate.
